# Effects of incorporation of granule-lyophilised platelet-rich fibrin into polyvinyl alcohol hydrogel on wound healing

**DOI:** 10.1038/s41598-018-32208-5

**Published:** 2018-09-19

**Authors:** Fangfang Xu, Dehui Zou, Taiqiang Dai, HaiYan Xu, Ran An, Yanpu Liu, Bin Liu

**Affiliations:** 1 State Key Laboratory of Military Stomatology & National Clinical Research Center for Oral Diseases &Shaanxi Clinical Research Center for Oral Diseases, Department of Oral and Maxillofacial Surgery, School of Stomatology, The Fourth Military Medical University, Xi’an, 710032 P.R. China; 2 Department of General Dentistry, Faculty of Stomatology, Xi’an Jiaotong University, Xi’an, 710004 China; 3 State Key Laboratory of Military Stomatology & National Clinical Research Center for Oral Diseases, Laboratory Animal Center, School of Stomatology, The Fourth Military Medical University, Xi’an, 710032 P.R. China

## Abstract

Dressings are commonly used to treat skin wounds. In this study, we aimed to develop a new scaffold composed of a polyvinyl alcohol (PVA) hydrogel containing granule-lyophilised platelet-rich fibrin (G-L-PRF) as a dressing. G-L-PRF was prepared by freeze-drying and was then incorporated into PVA hydrogel by freezing-thawing. Notably, the mechanical strength and degradation rate of the scaffold were found to be related to G-L-PRF concentrations, reaching 6.451 × 10^−2^ MPa and 17–22%, respectively, at a concentration of 1%. However, the strength decreased and the degradation was accelerated when the G-L-PRF concentration was over 1%. The elastic properties and biocompatibility of the scaffold were independent of G-L-PRF concentration, and both showed excellent elasticity and biocompatibility. The release of vascular endothelial growth factor and platelet-derived growth factor-AB was no significant time dependent. Additionally, application of 1% G-L-PRF/PVA to acute full-thickness dorsal skin wounds accelerated wound closure at days 7 and 9. Healing also increased on day 11. Histological and immunohistochemical analyses showed that the scaffold enhanced granulation tissue, maturity, collagen deposition, and new vessel formation. These results demonstrated that the prepared G-L-PRF/PVA scaffolds accelerated wound healing in acute full-thickness skin wounds, suggesting potential applications as an ideal wound dressing.

## Introduction

Full-thickness skin wounds caused by trauma, burns, and chronic diseases are serious conditions that are still challenging to treat^1^. Skin grafts and substitutes are the main treatment approaches for full-thickness skin wound. However, skin grafts can cause defects in the donor site and may not be the same colour and texture as the skin of the recipient. Moreover, grafts are limited by the timely^2^. Tissue-engineered skin may be an effective skin substitute, and some related products have become available; however, the risks of infection and rejection still exist^3–6^. Therefore, skin substitutes remain the first-line treatment.

Various scaffolds have been designed for the treatment of skin wounds, and such scaffolds have widely been used as skin substitutes. For example, chitosan is a natural linear cationic polysaccharide. It has good biocompatibility, biodegradability, and a antibacterial and hemostatic effects. Therefore, chitosan has been widely used in the production of composite dressings^7–9^. However, its obvious disadvantages are the presence of residual chemical additives and poor mechanical properties. Polyvinyl alcohol (PVA) is commonly used for the treatment of wounds because it is inexpensive, hydrophilic, highly elastic, transparent, and porous. Scaffolds have been designed using PVA-based hydrogels; those prepared with 10% PVA exhibit improved mechanical properties^10–12^. PVA hydrogels can be obtained by various methods, the most outstanding advantage over other materials is that it can be obtained through simple physical methods^13–15^.

The repair of wounds is complex and requires multiple materials^16^. Vascularisation is essential for wound healing, and biological base materials can simulate the extracellular matrix to repair skin damage; however, the use of a single substrate cannot induce sufficient blood vessel formation^17–19^. Numerous growth factors, such as vascular endothelial growth factor (VEGF), platelet-derived growth factor (PDGF), basic fibroblast growth factor (bFGF), transforming growth factor-β1, epidermal growth factor (EGF), and insulin-like growth factor, facilitate cell proliferation, differentiation, and neovascularisation. However, these growth factors are limited by their lack of sustained diffusion and degradation after direct injection into the body, and they are therefore often incorporated into controlled-release systems^20–25^. Wound healing is driven by different growth factors. However, controlled-release systems often encapsulate only one or two growth factors. Platelet-rich fibrin (PRF) is a new-generation platelet concentrate based on platelet-rich plasma^26–28^. This material is rich in a variety of growth factors, including VEGF, PDGF, bFGF, and EGF, which play key roles in tissue repair. Additionally, these growth factors can be released slowly. In our previous study, we found that the growth factors contained in PRF can be released continuously for more than 2 weeks. Other research has also confirmed that PRF is a more stable slow-release growth factor than PRP, which has been extensively studied in basic research and clinical applications^29–36^. As previously reported, the ability of PRF to promote regeneration is mainly due to the presence of a variety of growth factors. However, fresh PRF is not easy to store and transport, the elastic modulus is small, the hardness is low, and the immunogenicity is high. These problems limit the application of PRF in large-scale production.

Therefore, in this study, we attempted to develop a new dressing using PRF. We hypothesised that granule-lyophilised (G-L)-PRF/PVA could be prepared using a simple physical method in which fresh PRF was lyophilised and was then added to PVA hydrogels. The compound was transformed into a new dressing by freezing-thawing. To evaluate this new scaffold, the mechanical properties, cytotoxicity, cell viability, proliferation, and growth factor release were investigated *in vitro*. The healing properties were evaluated using acute full-thickness dorsal skin wounds created in normal mice.

## Results

### Morphology and ultrastructure of fresh PRF, L-PRF, and G-L-PRF/PVA scaffolds

**Figure 1:**
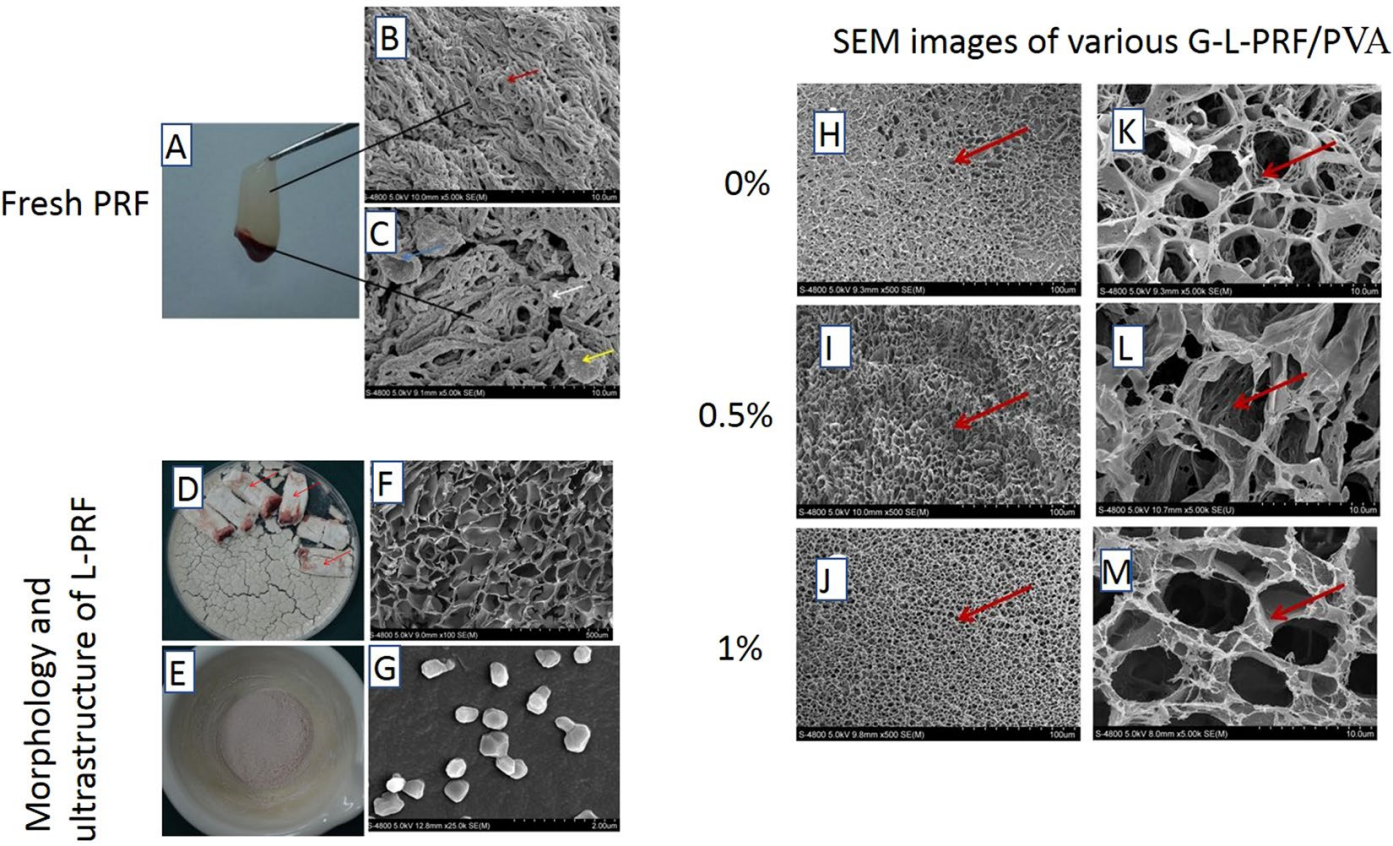
(**A**) Appearance of fresh PRF. (**B,C**) SEM images of fresh PRF. (**D,E**) Appearance of L-PRF and G-L-PRF. (**F,G**) SEM images of L-PRF and G-L-PRF. Red arrow = fibrin fibres, white arrow = platelets, yellow arrow = leukocytes, blue arrow = red blood cell. Morphology and ultrastructure of L-PRF are shown. SEM images of (**H,K**) the PVA group; (**I,L**) the 0.5% G-L-PRF/PVA group; (**J,K**) the 1% G-L-PRF/PVA group. The red arrow indicates the inner wall gap.

Fresh PRF was observed as a fibrin clot after centrifugation (Fig. 1A). Scanning electron microscopy (SEM) analysis revealed that there were no cells in the middle clot region (Fig. 1B), and platelets, leukocytes, and red blood cells were embedded in the border of yellow and red fibrin meshes (Fig. 1C).

L-PRF was present as a sponge (Fig. 1D, red arrow). SEM analysis showed that the surface had a porous shape (Fig. 1F). The ultrastructure of L-PRF after grinding and filtering (G-L-PRF, Fig. 1E) exhibited an ellipsoidal shape with a long axis of 118 ± 10.383 nm and a short axis of 81.65 ± 8.59 nm (Fig. 1G).

Analysis of the different concentrations of the G-L-PRF/PVA scaffold (Fig. 1) showed that at low magnification (500×), the surface morphology exhibited a continuous and interconnected network porous structure (Fig. 1H–J). Under higher magnification (3000×), the space between the inner wall showed that PVA without L-PRF was larger than those of 0.5% G-L-PRF/PVA and 1% G- L-PRF/PVA. In particular, the pores in 1% G-L-PRF/PVA were almost completely closed (Fig. 1K–M).

### Mechanical properties

At least three specimens for each group were recorded, and Table 1 presents the mechanical properties of the different specimens. As the G-L-PRF concentration increased in a certain range, the Young’s modulus decreased from 4.194 × 10^−2^ MPa for the PVA sample to 3.693 × 10^−2^ MPa for the 0.5% G- L-PRF/PVA sample; however, when the G-L-PRF concentration reached 1%, the Young’s modulus increased to 6.451 × 10^−2^ MPa, and when the concentration reached 2%, the Young’s modulus decreased to 3.40 × 10^−2^ MPa. The breaking elongation ratios of all samples indicated that the samples were excellent elastomers (ratios of about 300%), and, as shown in Fig. 4, there were no significant differences (*p* > 0.05).

**Table 1:** Mechanical properties of different groups.

Group	Young’s modulus (×10^−2^ MPa)	Elongation at break (%)
PVA	4.194 ± 0.601	312.733 ± 46.172
0.5% G-L-PRF/PVA	3.693 ± 1.076	302.604 ± 44.0456
1% G-L-PRF/PVA	6.451 ± 1.551	273.049 ± 24.510
2% G-L-PRF/PVA	3.40 ± 0.920	289.159 ± 35.671

### Biocompatibility

The scaffold and its leaching solution were analysed by MTT assays. In cocultures of L929 cells, there were no significant differences in absorbance values between the experimental group and the negative control (Table 2, *p* > 0.05), and the relative growth rate (RGR) was more than 88% (Table 2, toxicity level: 0–1) in the experimental group, but less than 38% (toxicity level: 3–4) in the positive control. The morphology of L929 cells in the 1% G-L-PRF/PVA group was similar to that of the blank group at the junction of the scaffold and the base plate, as demonstrated by light microscopy, fluorescence microscope, and SEM (see Supporting Information, Fig. S1).

**Table 2:** The A_490nm_ of dressing leaching solution determined by MTT assays (n = 5, mean ± standard deviation) and relative growth rate (RGR).

Group	A_490nm_	2 days		A_490nm_	4 days		A_490nm_	6 days	
		RGR (%)	Toxicity level		RGR (%)	Toxicity level		RGR (%)	Toxicity level
Negative	0.220 ± 0.009	100	0	0.596 ± 0.023	100	0	0.596 ± 0.023	100	0
Positive	0.083 ± 0.024	37.72%	3	0.019 ± 0.013	3.19%	4	0.019 ± 0.013	1.11%	4
PVA	0.199 ± 0.013	90.45%	1	0.527 ± 0.013	88.42%	1	0.527 ± 0.013	96.87%	1
0.25% G-L-PRF/PVA	0.203 ± 0.007	92.27%	1	0.601 ± 0.014	100.84%	0	0.601 ± 0.014	94.65%	1
0.5% G-L-PRF/PVA	0.227 ± 0.016	103.18%	0	0.546 ± 0.072	91.61%	1	0.546 ± 0.072	95.06%	1
0.75% G-L-PRF/PVA	0.206 ± 0.004	93.64%	1	0.562 ± 0.005	94.30%	1	0.562 ± 0.005	96.47%	1
1% G-L-PRF/PVA	0.201 ± 0.008	91.36%	1	0.532 ± 0.017	89.26%	1	0.532 ± 0.017	97.48%	1

### Biodegradation

**Figure 2:**
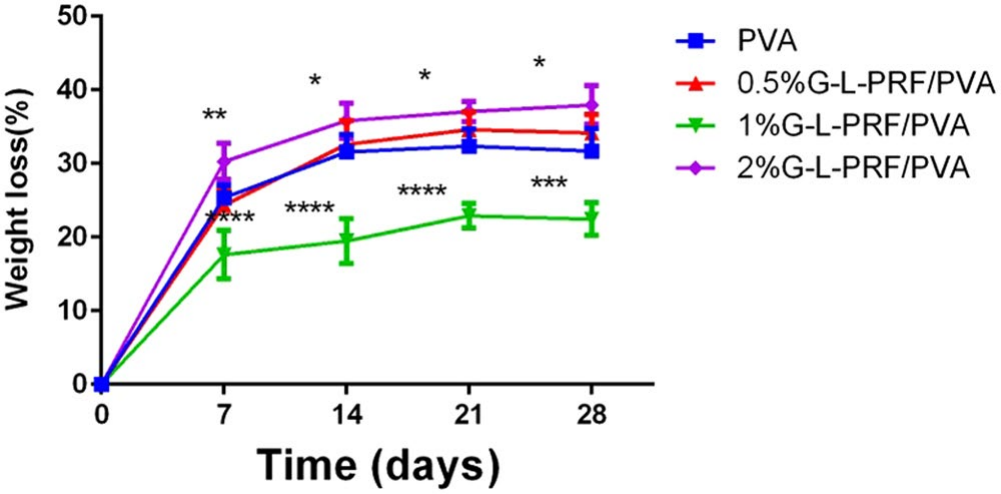
Weight loss in each group at 28 days (*P < 0.05, **P < 0.01, ***P < 0.001, ****P < 0.0001).

Figure 2 presents the biodegradability of G-L-PRF/PVA scaffolds with different concentrations of G-L-PRF. Although weight loss increased with embedment time, the degradation rates were 25–31% for the control group, 24–34% for 0.5% G-L-PRF/PVA, 17–22% for 1% G-L-PRF/PVA, and 30–37% for 2% G-L-PRF/PVA. The 1% group was significantly different from the other groups. However, changes in weight loss in the PVA and 0.5% G-L-PRF/PVA groups were not significant.

### Release of VEGF and PDGF-AB

**Figure 3:**
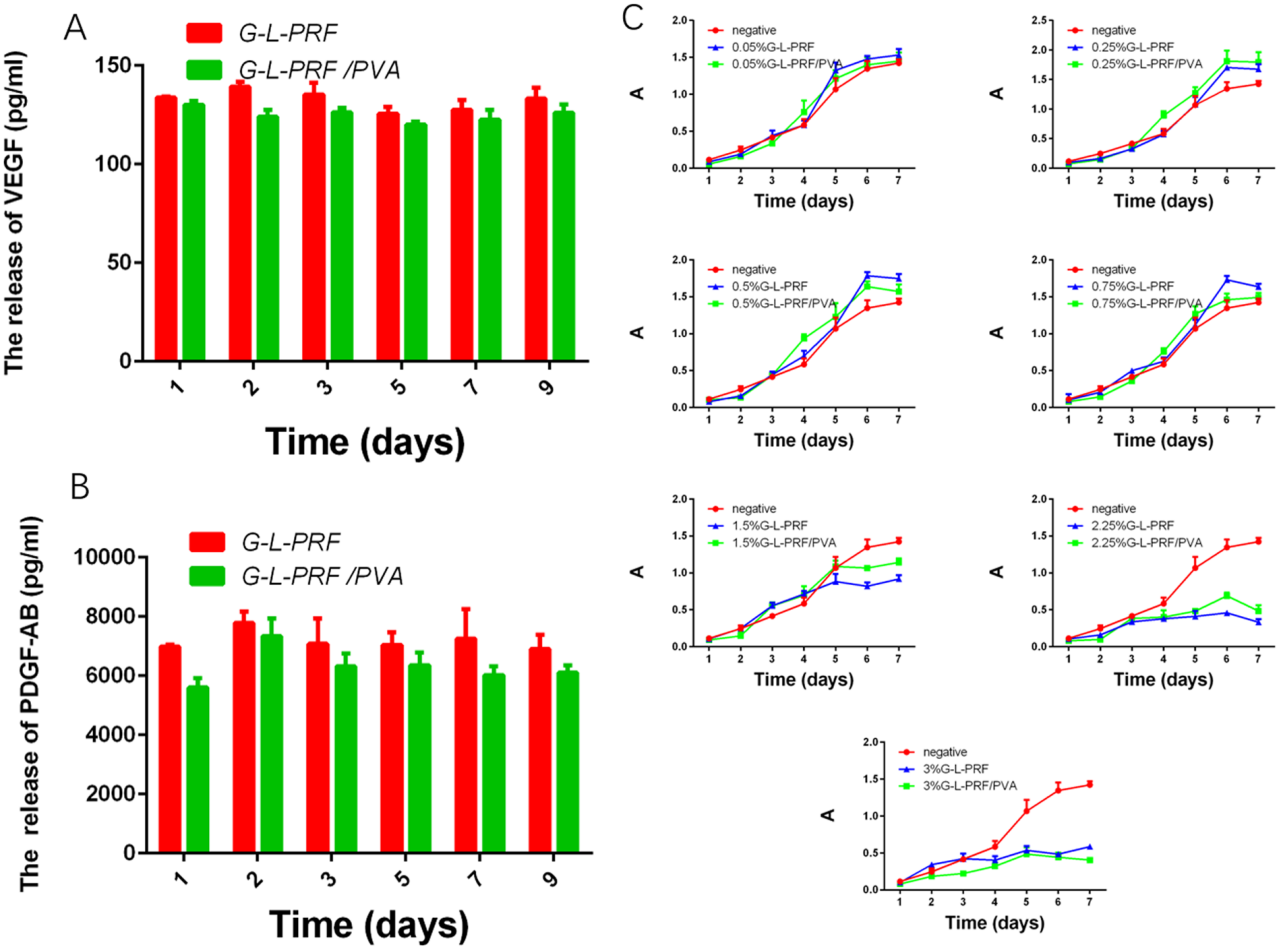
(**A,B**) The amount of released VEGF and PDGF-AB from G-L-PRF and G-L-PRF/PVA dressing leach liquor for 9 days. (**C**) Proliferation of HUVECs in the presence of different concentrations of L-PRF for days (absorbance value at 490 nm).

Next, we evaluated the concentrations of growth factors released from G-L-PRF and G-L-PRF/PVA scaffolds. Analysis of growth factor release (Fig. 3A,B) revealed that the amount of G-L-PRF/PVA released was less dependent on time than G-L-PRF. Thus, G-L-PRF/PVA permitted consistent and continuous release of growth factors.

### Evaluation of growth factor bioactivity

To elucidate the biological activities of the released growth factors, MTT assays were used to evaluate human umbilical vein endothelial cell (HUVEC) proliferation. Figure 3C presents the absorbance values for growth curves for the G-L-PRF and G-L-PRF/PVA groups, showing increased proliferation in the range of 0.05–0.75% G-L-PRF. However, when the G-L-PRF concentration was more than 1%, significantly reduced cell proliferation was observed in the G-L-PRF/PVA group. In contrast, in the G-L-PRF group, a concentration of 1.5% or more was needed for inhibition of cell proliferation.

### Animal test

Representative wound images obtained after treatment with the four scaffolds on days 3, 5, 7, 9, and 11 after wounding are shown in Supporting Information Fig. S2. The wounds of all treated mice were clean, and no infections were observed. By day 11, the 1% group showed a fully healed wound area, whereas small wounds were still visible in the other groups. Finally, wounds in the G-L-PRF group were healed on day 13, and wounds in the blank and PVA groups were healed on day 14. Therefore, the wound healing process was accelerated by the 1% G-L-PRF/PVA scaffold.

### Histological observations

**Figure 4:**
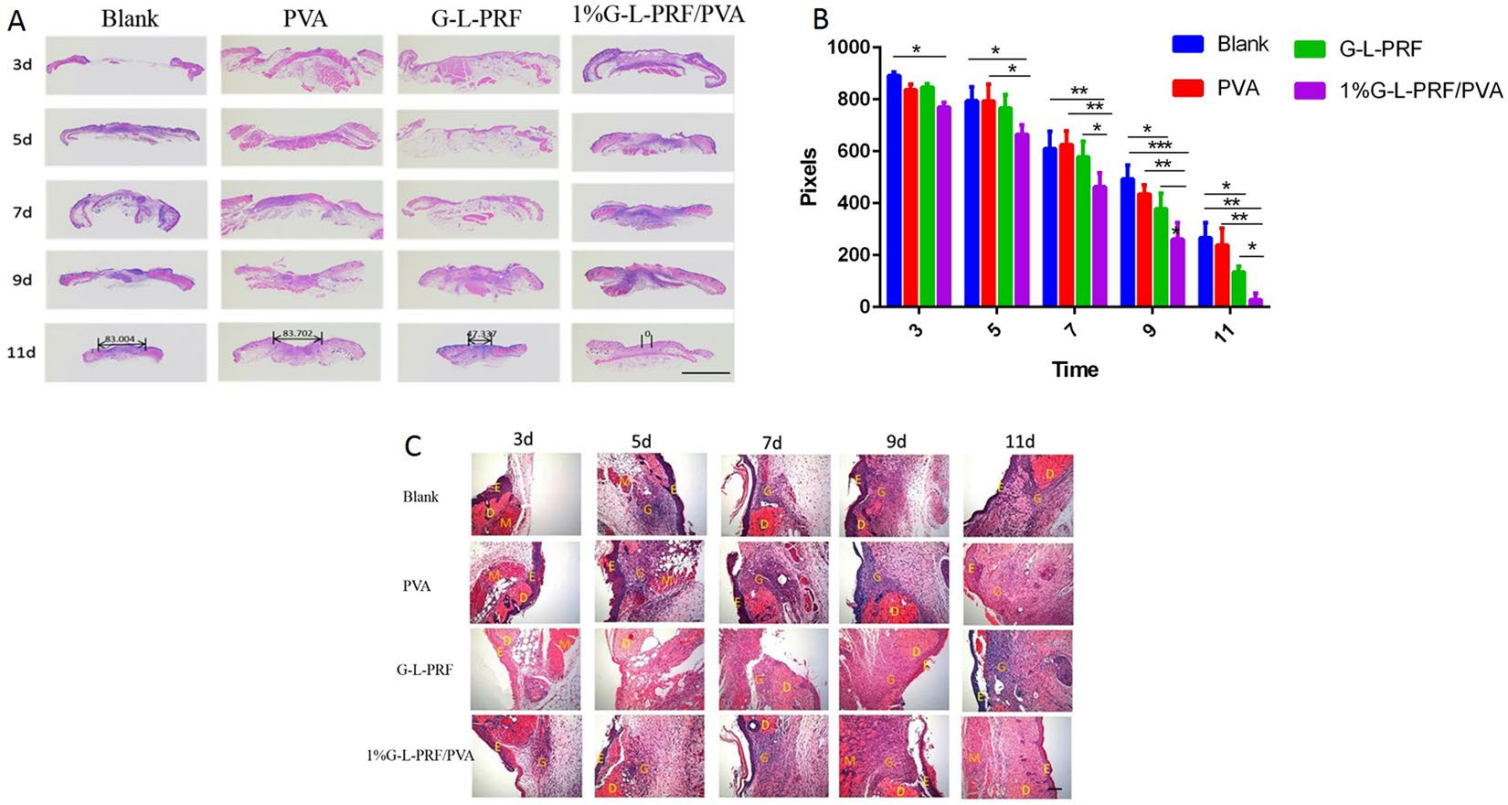
H&E staining of wounds in the blank, PVA, L-PRF, and 1% G-L-PRF/PVA groups for different days (scale bar in A = 2 mm). (**B**) The horizontal distance between the wound margin was measured using Image-Pro Plus 6.0 software. Bidirectional arrows indicate horizontal distances. (**C**) H&E images of sections after treatment with four groups at different time points. E = epithelium, D = dermis, G = granulation tissue, M = muscle (scale bar = 100 μm).

In the haematoxylin and eosin (H&E)-stained images (Fig. 4A), the boundary between the wound area and the normal skin was clear, and no tissue overlap was observed, indicating that the splint defect model was successful. The horizontal distance between the wound margin (Fig. 4A, black arrows) was measured using Image-Pro Plus 6.0 software (Fig. 4B). On days 3 and 5, 1% G-L-PRF/PVA scaffolds were significantly different from those in the blank group, even though the appearance was indistinguishable (Fig. 4B _3,5_; *P* < 0.05). Importantly, consistent with our general observations, there were significant differences between the 1% group and other groups on days 7 and 9, indicating that wound healing in the 1% group was maximised.

Representative images of dorsal skin wounds stained with H&E are shown in Fig. 4C. On day 3, only the 1% group exhibited some inflammatory tissues compared with the other groups. On days 5 and 7, the four groups showed different levels of inflammation. On day 9, the blank and PVA groups showed diffuse inflammatory cells in the wound area. However, very few inflammatory cells were found in the 1% group, and more granulation tissue was obvious; these changes were not observed in the G-L-PRF group. On day 11, all wounds were covered with new granulation tissue. The G-L-PRF group was mostly covered with epidermis, and a gap still existed between the newly formed tissues and the normal skin. In contrast, no gap was found in the 1% group, and the structure with skin appendages was similar to that of normal skin.

**Figure 5:**
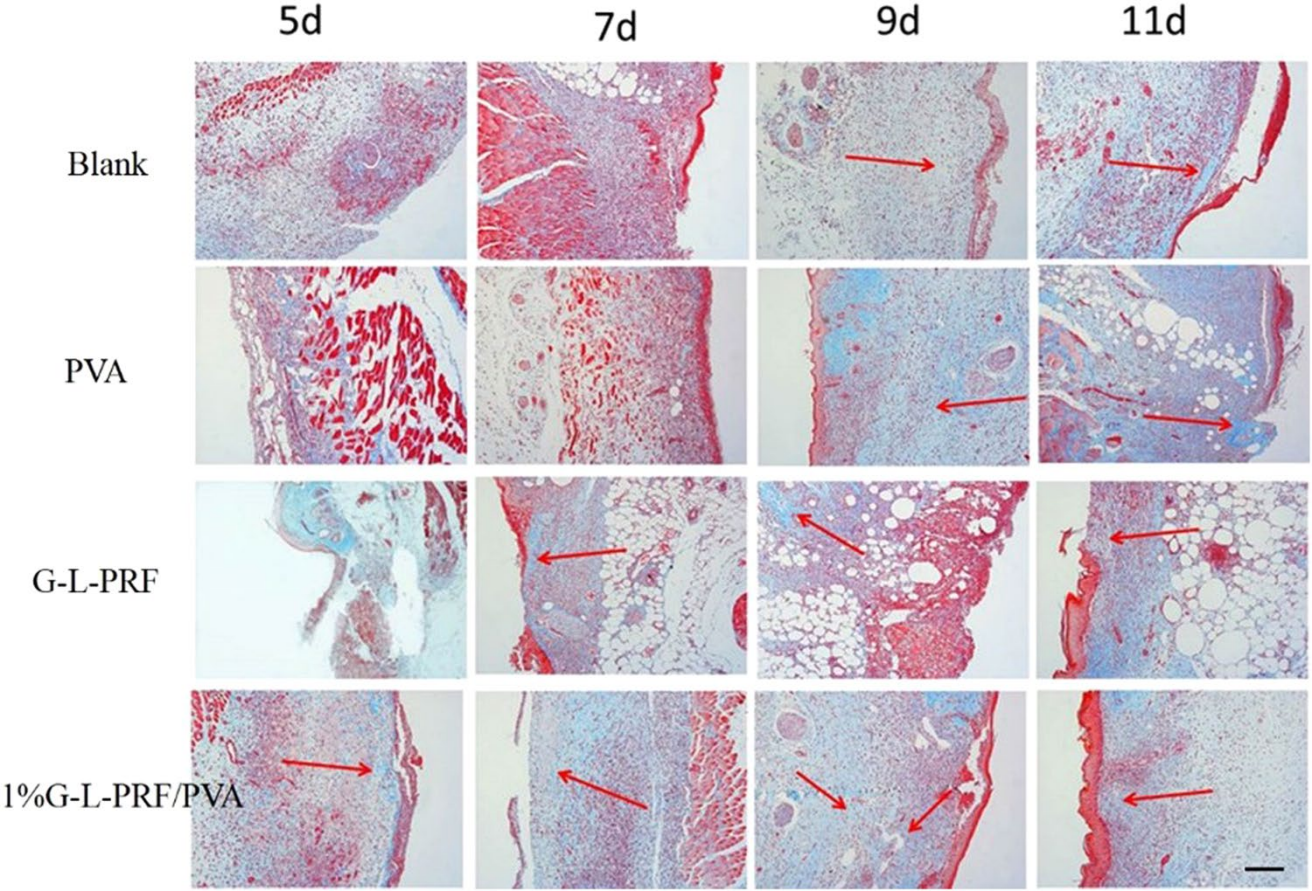
Masson’s trichrome staining images of sections after treatment with four groups at different time points. Red arrow, new collagen deposition (scale bar = 100 μm).

Collagen deposition was assessed by Masson’s trichrome staining for the different groups at the designated time points (Fig. 5). On day 5, no obvious collagen deposition was detected; however, some collagen fibres were detected in the 1% group. On day 7, the blank group exhibited thin collagen deposition, and the PVA and G-L-PRF groups showed slight collagen deposition; the 1% group exhibited small collagen fibres. On day 9, thick and thin collagen fibres were observed in the 1% group. In the G-L-PRF group, collagen deposition increased. Finally, on day 11, the collagen deposition in the 1% group was more uniform and regular than those in the other groups and resembled the normal dermal tissue. These results showed that new tissue formation was accelerated in the 1% group.

Picro-Sirius red staining was used to differentiate between different collagen types I and III, which are visualised as bright red and yellow. Our findings showed that thin and thick fibres were distributed in the deep dermis of the skin in the 1% group, appearing earlier than those in the other groups (Supporting Information, Fig. S3).

**Figure 6:**
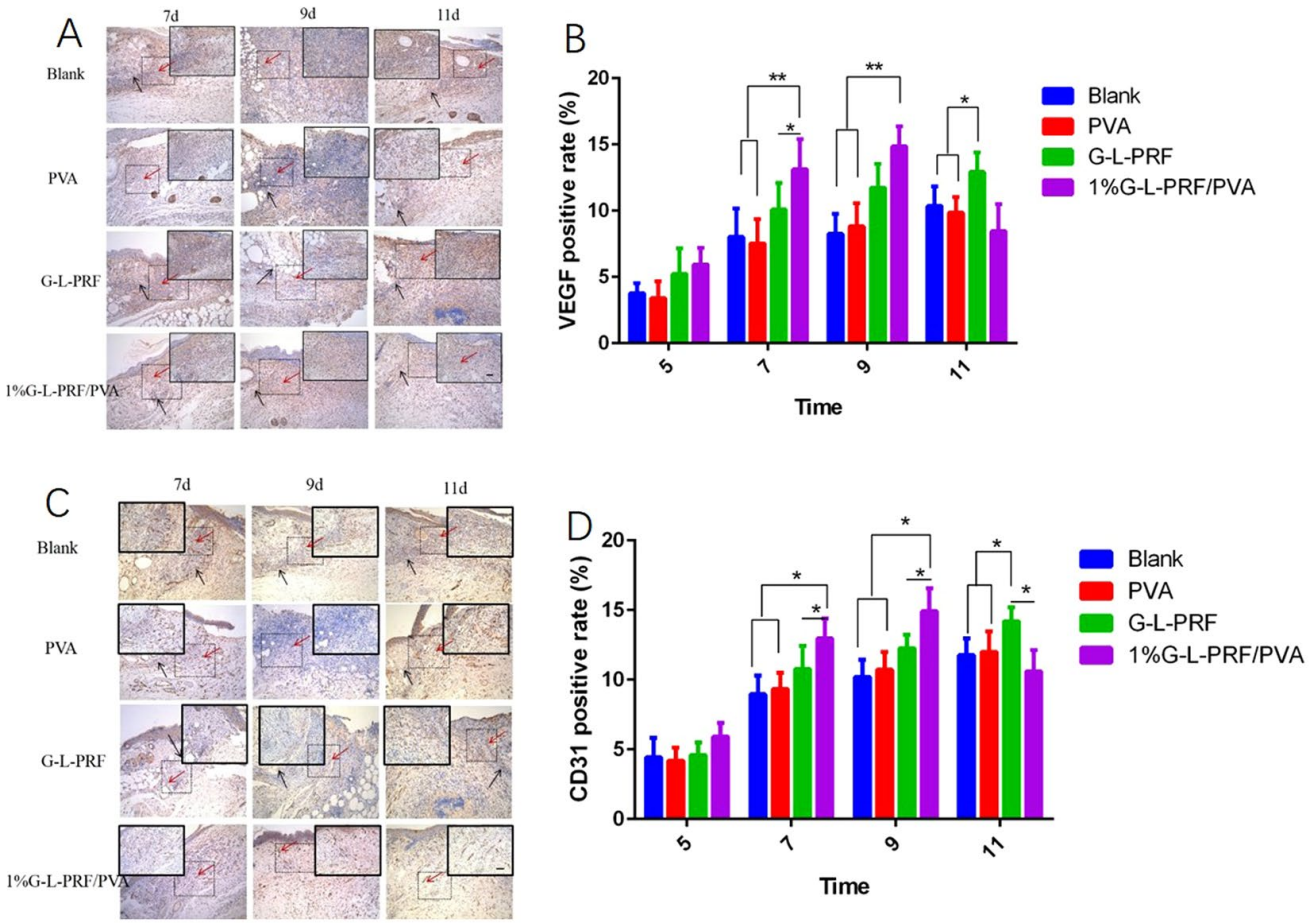
(**A**) VEGF immunohistochemical staining of sections of full-thickness wounds in the blank, PVA, L-PRF, and 1% G-L-PRF/PVA groups at different time points. Red arrows, positive expression of VEGF. Black arrows, normal tissues and wounds at the junction (scale bar = 50 μm). (**B**) VEGF expression in dressings was quantitatively assessed at different time points (*P < 0.05, **P < 0.01). (**C**) CD31 immunohistochemical staining of sections of full-thickness wounds in the blank, PVA, L-PRF, and 1% G-L-PRF/PVA groups at different time points. Red arrows, neovascularisation. Black arrows, normal tissues and wounds at the junction (scale bar = 50 μm). (**D**) CD31 expression in dressings was quantitatively assessed at different time points (*P < 0.05).

To examine the presence of VEGF in the four wound treatment groups, immunohistochemical staining was performed (Fig. 6A). On days 7 and 9, VEGF was significantly upregulated in the regenerated tissues (normal tissue and wound at the junction) of the 1% group compared with that on the other groups. Additionally, the G-L-PRF group showed more abundant expression than the other groups. On day 14, VEGF was still expressed in all groups, but decreased in the 1% group. As shown in Fig. 6B, the expression levels of VEGF were higher than those in the 1% group on days 7 and 9 and then decreased on 11 day.

Newly formed vessels in the wounds were characterised by CD31 staining (Fig. 6C), and the positive rate was calculated (Fig. 6D). At all time points, the CD31-positive rate increased, except in the 1% group on day 11. Specifically, on days 7 and 9, the positive rate in the 1% group was significantly higher than those in the other groups. On day 11, the CD31 rate in regenerated tissues decreased gradually (initial rate: 10.60%); however, that in the G-L-PRF group had increased to 14.21%, which was higher than those of the other groups.

## Discussion

Various wound scaffolds have been used to treat skin defects^3,4,16^. Wound scaffolds are not just simple covers; they should also have the following properties: biocompatibility, no immunogenicity, barrier functionality, moisturising effects, and low cost^37^. In this study, G-L-PRF was loaded into PVA hydrogels at 45 ± 5 °C by magnetic stirring and a modified freeze-thaw methods (with a single time cycle) in order to protect growth factors produced by G-L-PRF from inactivation by the high temperature and repeated freeze-thaw cycles. We demonstrated that G-L-PRF/PVA provided sustained, controlled release of VEGF and PDGF using HUVECs^24,30^. Moreover, angiogenesis can be enhanced by various factors, including VEGF and PDGF, and sustained release of these factors by a delivery system is necessary for wound healing^20,24,25^. In our study, we found that increasing the concentration of G-L-PRF in the PVA hydrogels also increased the Young’s modulus, decreased the inner wall pore size of G-L-PRF/PVA, and reduced biodegradation. However, when the concentration was more than 1%, the Young’s modulus and HUVEC viability decreased. Additionally, regardless of changes in the concentration of G-L-PRF, the breaking elongation ratio remained around 300%, and no cytotoxicity was observed. Therefore, our results suggested that G-L-PRF may have an effect on PVA internal termolecular crosslinks and morphology^38,39^. The morphology of G-L-PRF/PVA scaffolds changed at different concentrations, demonstrating that G-L-PRF affected PVA crosslinks. Mechanical strength and elasticity are important for scaffolds^40,41^ and for maintaining the minimum required strength during wound healing; hence, the scaffold should slowly degrade into small molecules^41^. The results indicated that 1% G-L-PRF/PVA scaffolds showed good mechanical strength and excellent elastomer properties, which were suitable for application as wound scaffolds.

Wound repair is highly complex, and animal models are needed to simulate human wound healing^42^. Splint wounds in mouse models of acute full-thickness skin wounds can not only uncover important pathways and processes but also avoid contraction during wound healing^43,44^. In our study, the healing potential of the 1% G-L-PRF/PVA scaffolds was investigated in a mouse model of acute wound defects. As the time after operation increased, the wound size decreased; however, macroscopic evaluation indicated that wounds covered with 1% G-L-PRF/PVA healed more rapidly. H&E staining, Masson’s trichrome staining, and Picro-Sirius red staining of cells are usually performed, and collagen fibres and maturity are evaluated at different time points after surgery^45,46^. The G-L-PRF/PVA group showed low inflammatory cell infiltration, which could activate cell migration and proliferation, resulting in neovascularisation and neotissue formation during the early stages of skin repair^47^. Therefore, the deposition thickness, regularity, and maturity of fresh collagen in the G-L-PRF/PVA group were significantly better than those in the other groups at defect sites. Furthermore, the new collagen deposition was similar to the normal dermal tissue, indicating that the 1% G-L-PRF/PVA had high biological activity. CD31 and VEGF were used to evaluate the newly formed vessels^18,48^. Vascularisation in the 1% G-L-PRF/PVA group was accelerated during the wound-healing processes, and higher expression levels were observed in this group compared with those in the other groups before 9 days after surgery. Therefore, the newly formed blood vessels could supply oxygen and nutrients, thereby facilitating collagen synthesis. However, at day 11 after surgery, the expression levels of VEGF and CD31 were lower than those in the other groups, demonstrating downregulation of growth factors to form normal skin tissues^49,50^.

In conclusion, the current study demonstrated that 1% G-L-PRF/PVA had excellent mechanical and biological activity. Although other important issues remain to be addressed, such as the fate of the degraded materials in the body, the current study provided an ideal scaffold for promotion of healing in skin wounds.

## Materials and Methods

### Preparation of G-L-PRF and G-L-PRF/PVA scaffolds

Fresh PRF was prepared using a simple technique as reported previously^36^. Briefly, blood samples were collected from rabbit ear arteries and placed into 10-mL sterile glass tubes. The tubes were immediately centrifuged at 400 × *g* for 10 min. The yellow part of the fibrin gel was easily separated from the red part using sterile stainless steel scissors. The fresh PRF was lyophilised (Virtis, USA) to produce L-PRF. The samples were then ground into granules using a mortar. The granules were filtered through a screen mesh (pore size: 0.076 mm), producing G-L-PRF. PVA hydrogels (Guoyao Chemicals, China; polymerisation degree: 1750 ± 50, hydrolysis degree: 99%) were prepared following the procedure described previously^14^. Briefly, PVA hydrogels at a concentration 10% w/v were prepared by dissolving PVA in purified water at 90 °C under magnetic stirring for 3 h. The samples were then slowly cooled to 45 °C ± 5 °C, and 0.5, 0.75, 1, or 2 g G-L-PRF was added to PVA hydrogels by stirring for 1 h to obtain well-distributed hydrogels. The complexes were poured into 12- or 24-well plates (Corning, USA) at 0.5 mL/well. The plates were then frozen for 24 h at −20 °C and thawed for 24 h at room temperature. This procedure was repeated only one time, yielding G-L-PRF/PVA hydrogels.

### SEM observations

SEM was used to identify the morphologies of fresh PRF, L-PRF, and G-L-PRF/PVA. Before SEM observation, fresh PRF specimens were fixed with a solution of 4% paraformaldehyde at 4 °C for 1 h and then dehydrated using a graded ethanol series^35^. The L-PRF was placed in ethanol (75%) by stirring to obtain a well-distributed suspension and then dripped onto cover glass. All specimens were examined by SEM (S-4800; Hitachi, Japan).

### Mechanical properties

G-L-PRF/PVA was cut into the same dumbbell shape (approximately 20 mm × 5 mm × 0.2 mm), and the length, width, and thickness were measured using a micrometer. The mechanical properties of specimens were evaluated at room temperature using a universal testing machine (EZ-Test500N, Japan). During measurement, the specimens were pulled at a rate of 10 mm/min. The Young^’^s modulus and elongation at break were calculated as follows^52^:

$${\rm{Young}}\mbox{'}{\rm{s}}\,{\rm{modulus}}=\frac{{\rm{F}}\times {\rm{L}}}{{\rm{A}}\times {\rm{\Delta }}{\rm{L}}}$$

$${\rm{Elongation}}\,{\rm{at}}\,{\rm{break}}( \% )=\frac{{\rm{\Delta }}{\rm{L}}}{{\rm{L}}}\times 100 \% $$

where F is the breaking force (N), L is the original length (mm), ΔL is the increase in length at breaking, and A is the cross-sectional area (mm^2^).

### *In vitro* cytotoxicity

L929 cells were cultured in complete medium (Dulbecco’s modified Eagle’s medium with 100 U/mL penicillin/streptomycin; Hyclone, USA) under a humid atmosphere of 95% air and 5% CO_2_ at 37 °C. The medium was replaced every other day. The cells were used for analysis of cytotoxicity. G-L-PRF and G-L-PRF/PVA scaffolds (30 mg/mL) were sterilised using ultraviolet light and then placed into sterile centrifuge tubes containing complete medium at 37 °C in a humidified atmosphere of 5% CO_2_. After 24 h, the tubes were centrifuged at 1000 rpm for 5 min and filtered (0.22-mm filter). The leaching solution was used for cytotoxicity analysis.

The G-L-PRF/PVA scaffolds and PVA hydrogels were cut into slices and sterilised using ultraviolet light. L929 cells (1 × 10^5^ cells/mL) were seeded on the scaffolds and hydrogels slices in 96-well plates (200 µL/well) and incubated in a humid atmosphere of 95% air and 5% CO_2_ at 37 °C for 24 h. Cell adhesion and morphology on the scaffolds and hydrogels were characterised by light microscopy, fluorescent microscopy, and SEM. Briefly, the culture medium was removed by aspiration, and cells were rinsed with phosphate-buffered saline gently. Cells were then fixed with 2.5% glutaraldehyde at 4 °C for 8 h, and some of the samples were dehydrated in ethanol solutions of various concentrations (30%, 50%, 70%, 80%, 90%, 95%, and 100%), followed by sputter-coating with a thin gold layer for SEM analysis. Other samples were analysed using rabbit anti-waveform silk protein antibodies, incubated overnight at 4 °C, and then treated with IgG/fluorescein isothiocyanate and 4′,6-diamidino-2-phenylindole for observation by fluorescence microscopy (Olympus IX71, Japan).

### Release of VEGF and PDGF-AB

Growth factors from L-PRF and G-L-PRF/PVA, such as PDGF-AB and VEGF, were quantified using enzyme-linked immunosorbent assays (ELISAs; R&D Systems, Shanghai, China) according to the manufacturer’s instructions. Briefly, L-PRF (30 mg) and G-L-PRF/PVA scaffolds (with 30 mg L-PRF) were placed in a centrifuge tube containing 1 mL serum-free medium (Hyclone) and incubated at 37 °C in a humidified atmosphere containing 5% CO_2_. During culture, the medium was collected after 1, 2, 3, 5, 7, or 9 days, and all collected samples were stored in 2-mL cryovials at −80 °C before analysis.

### Evaluation of the bioactivities of growth factors

The bioactivities of growth factors released from G-L-PRF/PVA scaffolds were determined *in vitro* by assessing their ability to stimulate HUVEC proliferation. Briefly, HUVECs were seeded in 96-well plates at a density of 4 × 10^4^ cells/mL with the collected medium from the G-L-PRF/PVA scaffolds. After incubation for 1, 2, 3, 4, 5, 6, or 7 days, the cell number was determined by MTT assays.

### *In vivo* biodegradation

The biodegradability assay was carried out to measure the biodegradation of PVA hydrogels and G-L-PRF/PVA scaffolds. To simulate the environment of the human body, the C_57_ mice (obtained from the Animal Center, Fourth Military Medical University; age 6–8 weeks, weighing 20–25 g) were divided into four groups (n = 12 per group): PVA hydrogel alone, 0.5% G-L-PRF/PVA, 1% G-L-PRF/PVA, and 2% G-L-PRF/PVA. Briefly, mice were anaesthetised, and dorsal surface hair was shaved. An incision was then created using a scalpel (width: 1 cm, depth: 1 cm). Two incisions were made, one on each side of the midline. The specimens were then placed in the incision, and the incisions were closed using sutures. The mice were sacrificed after 1, 2, 3, or 4 weeks using an overdose of anaesthetic. The weight lost by the sample was measured each time. The biodegradability was calculated as follows;

$${\rm{Biodegradability}}( \% )=\frac{{\rm{\Delta }}{\rm{G}}}{{\rm{G}}}\times 100 \% $$

where ΔG is the weight change, and G is the weight before degradation.

### Animal test in a mouse model

Animal experimental manipulation were carried out according to local animal welfare guidelines and regulations, and were approved by the School of Stomatology, Fourth Military Medical University (ID: kq-033)

In this experiment, C_57_ mice (the Laboratory Animal Center, the Fourth Military Medical University, Xi’an, China) were divided into four groups (n = 15 per group): blank, PVA hydrogel, L-PRF, and 1% G-L-PRF/PVA. The healing properties of the G-L-PRF/PVA scaffolds on acute full-thickness skin defects were then studied^45^. Briefly, C_57_ mice were anaesthetised by intraperitoneal injection of pentobarbital sodium solution (75 mg/kg). Before surgery, the dorsal surface was shaved with an electric clipper and 8% Na_2_S to remove any remaining hair. Mice were then sterilised with betadine and alcohol. Two full-thickness incisional wounds with a diameter of 6 mm were made using a puncher on the dorsal region. A donut-shaped splint with an internal diameter of 10 mm and an external diameter of 12 mm was placed such that the wound was centred within the splint to inhibit natural skin shrinkage. The four samples were then applied to the wounds, and Tegader Film (3 M, 1624W, USA) was used to cover the scaffold to improve adherence. Digital photographs were taken of the tissue samples, and samples were harvested at 3, 5, 7, 9, and 11 days after surgery.

### Histology and immunohistochemistry

For histological analyses, the harvested samples were fixed in paraformaldehyde (4%), dehydrated with a graded series of ethanol concentrations, and embedded in paraffin. Sectioned samples with a thickness of 4–6 mm were then stained with H&E, Masson, and Picro-Sirius red staining^46,51^. To study angiogenesis during wound healing, two key factors related to angiogenesis (VEGF and CD31) were detected by immunohistochemical staining.

### Statistical analysis

All vitro experiments were replicated at least three times. In animal studies, the images of macroscopic and microscopic fields from animals were analysed using Image-Pro Plus 6.0 (USA). Data are expressed as means ± standard deviations, and statistical significance was determined using one-way analysis of variance, followed by Newman-Keuls test (SNK-q). Differences with *p* values of less than 0.05 were considered significant. Statistical analysis was performed using SPSS 17.0 software (SPSS, USA) with GraphPad Prism 6.0 (USA).

## Electronic supplementary material

supplementary information

## Data Availability

All data generated during this study are included in this article (and included Supplementary Information files).

## References

[1] Hrabchak C, Flynn L, Woodhouse KA (2006). Biological Skin Substitutes for Wound Cover and Closure. Expert Rev Med Devices.

[2] Guo R, Xu S, Ma L, Huang A, Gao C (2010). Enhanced Angiogenesis of Gene-Activated Dermal Equivalent for Treatment of Full Thickness Incisional Wounds in a Porcine Model. Biomaterials.

[3] Ghosal, K., Manakhov, A., Zajíčková, L. & Thomas, S. Structural and Surface Compatibility Study of Modified Electrospun Poly(Ε-Caprolactone) (PCL) Composites for Skin Tissue Engineering. *Aaps Pharmscitech*. (2016). 10.1208/s12249-016-0500-8 26883261

[4] Auger FA (1988). Tissue-engineered human skin substitutes developed from collagen-populated hydrated gels: clinical and fundamental applications. Med Biol Eng Comput.

[5] Chong AK, Chang J (2006). Tissue Engineering for the Hand Surgeon: A Clinical Perspective. J Hand Surg Am.

[6] Nathoo R, Howe N, Cohen G (2014). Skin Substitutes: An Overview of the Key Players in Wound Management. J Clin Aesthet Dermatol.

[7] Ishihara M (2006). Chitosan Hydrogel as a Drug Delivery Carrier to Control Angiogenesis. J Artif Organs.

[8] Mohandas A, Anisha BS, Chennazhi KP, Jayakumar R (2015). Chitosan–Hyaluronic acid/VEGF Loaded Fibrin Nanoparticles Composite Sponges for Enhancing Angiogenesis in Wounds. Colloids and Surfaces B: Biointerfaces.

[9] Hong H, Jin S, Park J, Ahn WS, Kim C (2008). Accelerated Wound Healing by Smad3 Antisense Oligonucleotides-Impregnated Chitosan/Alginate Polyelectrolyte Complex. Biomaterials.

[10] Stasko J, Kalniņš M, Dzene A, Tupureina V (2009). Poly(Vinyl Alcohol) Hydrogels. P Est Acad Sci.

[11] Li X, Hu A, Ye L (2011). Structure and Property of Porous Polyvinylalcohol Hydrogels for Microorganism Immobilization. J POLYM ENVIRON.

[12] Zhang SD, Zhai YC, Zhang ZF (2011). Preparation and Properties of PolyvinylAlcohol (PVA)/Polyvinyl Pyrrolidone (PVP) Hydrogel. Applied Mechanics & Materials.

[13] Purss HK, Qiao GG, Solomon DH (2005). Effect of “Glutaraldehyde” Functionality On Network Formation in Poly (Vinyl Alcohol) Membranes. J Appl Polym Sci.

[14] Amin MA, Abdel-Raheem IT (2014). Accelerated Wound Healing and Anti-Inflammatory Effects of Physically Cross Linked Polyvinyl Alcohol–Chitosan Hydrogel Containing Honey Bee Venom in Diabetic Rats. Arch Pharm Res.

[15] Ajji Z (2005). Preparation of poly(vinyl alcohol) hydrogels containing citric or succinic acid using gamma radiation. Radiation Physics & Chemistry.

[16] Liu, Q. *et al*. Acceleration of Skin Regeneration in Full-Thickness Burns by Incorporation of bFGF-loaded Alginate Microspheres Into a CMCS-PVA Hydrogel. *J Tissue Eng Regen Med*. (2015). 10.1002/term.2057 26118827

[17] Folkman J, Shing Y (1992). Angiogenesis. J Biol Chem.

[18] Novosel EC, Kleinhans C, Kluger PJ (2011). Vascularization is the Key Challenge in Tissue Engineering. Adv Drug Deliv Rev.

[19] Mao J (2003). Study of Novel Chitosan-Gelatin Artificial Skin *in Vitro*. J Biomed Mater Res A.

[20] Losi P (2013). Fibrin-Based Scaffold Incorporating VEGF- and bFGF-loaded Nanoparticles Stimulates Wound Healing in Diabetic Mice. Acta Biomater.

[21] Pierce GF (1992). Platelet-Derived Growth Factor (BB Homodimer), Transforming Growth Factor-Beta 1, and Basic Fibroblast Growth Factor in Dermal Wound Healing. Neovessel and Matrix Formation and Cessation of Repair. Am J Pathol.

[22] Spiller KL (2012). A Novel Method for the Direct Fabrication of Growth Factor-Loaded Microspheres within Porous Nondegradable Hydrogels: Controlled Release for Cartilage Tissue Engineering. J Control Release.

[23] Ulubayram K, Nur CA, Korkusuz P, Ertan C, Hasirci N (2001). EGF Containing Gelatin-Based Wound Dressings. Biomaterials.

[24] Losi P (2010). Tissue Response to Poly(Ether)urethane-Polydimethylsiloxane-Fibrin Composite Scaffolds for Controlled Delivery of Pro-Angiogenic Growth Factors. Biomaterials.

[25] Bourke SL (2003). A Photo-Crosslinked Poly(Vinyl Alcohol) Hydrogel Growth Factor Release Vehicle for Wound Healing Applications. AAPS PharmSci.

[26] Dohan DM (2006). Platelet-Rich Fibrin (PRF): A Second-Generation Platelet Concentrate. Part I: Technological Concepts and Evolution. Oral Surg Oral Med Oral Pathol Oral Radiol Endod.

[27] Choukroun J (2006). Platelet-Rich Fibrin (PRF): A Second-Generation Platelet Concentrate. Part IV: Clinical Effects On Tissue Healing. Oral Surg Oral Med Oral Pathol Oral Radiol Endod.

[28] Anitua E (2006). New Insights Into and Novel Applications for Platelet-Rich Fibrin Therapies. Trends Biotechnol.

[29] Choukroun J (2006). Platelet-Rich Fibrin (PRF): A Second-Generation Platelet Concentrate. Part V: Histologic Evaluations of PRF Effects On Bone Allograft Maturation in Sinus Lift. Oral Surgery, Oral Medicine, Oral Pathology, Oral Radiology, and Endodontology.

[30] Schär MO, Diaz-Romero J, Kohl S, Zumstein MA, Nesic D (2015). Platelet-Rich Concentrates Differentially Release Growth Factors and Induce Cell Migration *in Vitro*. Clinical Orthopaedics and Related Research®.

[31] Mohanty S, Pathak H, Dabas J (2014). Platelet Rich Fibrin: A New Covering Material for Oral Mucosal Defects. Journal of Oral Biology and Craniofacial Research.

[32] Nagaveni NB, Poornima P, Joshi JS, Pathak S, Nandini DB (2015). Revascularization of Immature, Nonvital Permanent Tooth Using Platelet-Rich Fibrin in Children. Pediatr Dent.

[33] Yuanzheng C (2015). Enhancement of the Repair of Dog Alveolar Cleft by an Autologous Iliac Bone, Bone Marrow–Derived Mesenchymal Stem Cell, and Platelet-Rich Fibrin Mixture. Plast Reconstr Surg.

[34] Bains V, Gupta V, Jhingran R, Mathur A, Singh GP (2015). Evaluation of Intrabony Defects Treated with Platelet-Rich Fibrin Or Autogenous Bone Graft: A Comparative Analysis. European Journal of Dentistry.

[35] Zhao YH (2013). The Combined Use of Cell Sheet Fragments of Periodontal Ligament Stem Cells and Platelet-Rich Fibrin Granules for Avulsed Tooth Reimplantation. Biomaterials.

[36] Liu B (2012). The Adjuvant Use of Stromal Vascular Fraction and Platelet-Rich Fibrin for Autologous Adipose Tissue Transplantation. Tissue Engineering Part C Methods.

[37] Schulz, A. *et al*. A Prospective Clinical Trial Comparing Biobrane® Dressilk® and PolyMem® Dressings On Partial-Thickness Skin Graft Donor Sites. *BURNS* (2015). 10.1016/j.burns.2014.12.016 25720659

[38] Hassan CM, Peppas NA (2000). Structure and Morphology of Freeze/Thawed PVA Hydrogels. Macromolecules.

[39] Yang Y (2014). Structural Insights Into Enzymatic Degradation of Oxidized Polyvinyl Alcohol. Chembiochem.

[40] Hansen B, Jemec GB (2002). The Mechanical Properties of Skin in Osteogenesis Imperfecta. Arch Dermatol.

[41] Hsieh W, Liau J (2013). Cell Culture and Characterization of Cross-Linked Poly(Vinyl Alcohol)-G-Starch 3D Scaffold for Tissue Engineering. Carbohyd Polym.

[42] Ansell DM, Holden KA, Hardman MJ (2012). Animal Models of Wound Repair: Are they Cutting It?. Exp DermatoL.

[43] Davidson JM (1998). Animal Models for Wound Repair. Arch Dermatol Res.

[44] Galiano RD, Michaels JT, Dobryansky M, Levine JP, Gurtner GC (2004). Quantitative and Reproducible Murine Model of Excisional Wound Healing. WOUND REPAIR REGEN.

[45] Yang, Y. *et al*. Promotion of Skin Regeneration in Diabetic Rats by Electrospun Core-Sheath Fibers Loaded with Basic Fibroblast Growth Factor **32**, 4243–4254 (2011). 10.1016/j.biomaterials.2011.02.042 21402405

[46] Prabhu V (2014). Objective Assessment of Endogenous Collagen *in Vivo* During Tissue Repair by Laser Induced Fluorescence. Plos One.

[47] Yan S (2013). Silk Fibroin/Chondroitin Sulfate/Hyaluronic Acid Ternary Scaffolds for Dermal Tissue Reconstruction. Acta Biomater.

[48] Davies N (2008). The Dosage Dependence of VEGF Stimulation On Scaffold Neovascularisation. Biomaterials.

[49] Wang DI, Gotlieb AI (1999). Fibroblast Growth Factor 2 Enhances Early Stages of *in Vitro* Endothelial Repair by Microfilament Bundle Reorganization and Cell Elongation. Exp Mol Pathol.

[50] Kouchak M, Ameri A, Naseri B, Kargar BS (2014). Chitosan and Polyvinyl Alcohol Composite Films Containing Nitrofurazone: Preparation and Evaluation. Iran J Basic Med Sci.

[51] Junqueira LC, Cossermelli W, Brentani R (1978). Differential Staining of Collagens Type I, II and III by Sirius Red and Polarization Microscopy. Arch Histol Jpn.

