# Supplementary material for: Effects of incorporation of granule-lyophilised platelet-rich fibrin into polyvinyl alcohol hydrogel on wound healing
Source: Sci Rep. 2018 Sep 19;8:14042. doi: 10.1038/s41598-018-32208-5 (PMC6145885; doi:10.1038/s41598-018-32208-5)
Supplement: Supplementary file 1 — supplementary information [file 41598_2018_32208_MOESM1_ESM.pdf]

Effects of incorporation of granule-lyophilised platelet-rich fibrin into polyvinyl alcohol hydrogel on wound healing

Fangfang Xu, Ph.D.[a,#], Dehui Zou, M.D.[b, #], Taiqiang Dai, Ph.D.[a] , HaiYan Xu, M.S.[C], Ran An, M.S.[C], Yanpu Liu, D.D.S., Ph.D.[a,*], Bin Liu, D.D.S., Ph.D.[C,*]

[a]State Key Laboratory of Military Stomatology & National Clinical Research Center for Oral Diseases &Shaanxi Clinical Research Center for Oral Diseases, Department of Oral and Maxillofacial Surgery, School of Stomatology, The Fourth Military Medical University, Xi'an 710032, P.R. China

[b]Department of General Dentistry, Faculty of Stomatology, Xi'an Jiaotong University, Xi'an 710004, China

[c]State Key Laboratory of Military Stomatology & National Clinical Research Center for Oral Diseases, Laboratory Animal Center, School of Stomatology, The Fourth Military Medical University, Xi'an 710032, P.R. China

[#]These authors contributed equally to this work and should be considered co-first authors

***Corresponding authors:**

Bin Liu, Professor

State Key Laboratory of Military Stomatology, Laboratory Animal Center, School of Stomatology, the Fourth Military Medical University, 145 West Changle Road, Xi'an 710032, P. R. China; Tel: +86-29-84776175; Fax: +86-29-84776175; E-mail: kqyljd_liu@126.com

Yanpu Liu, Professor

State Key Laboratory of Military Stomatology, Department of Oral and Maxillofacial Surgery, School of Stomatology, the Fourth Military Medical University, 145 West Changle Road, Xi'an 710032, P. R. China; Tel: +86-29-84772532; Fax: +86-29-83224470; E-mail: liuyanpu@fmmu.edu.cn

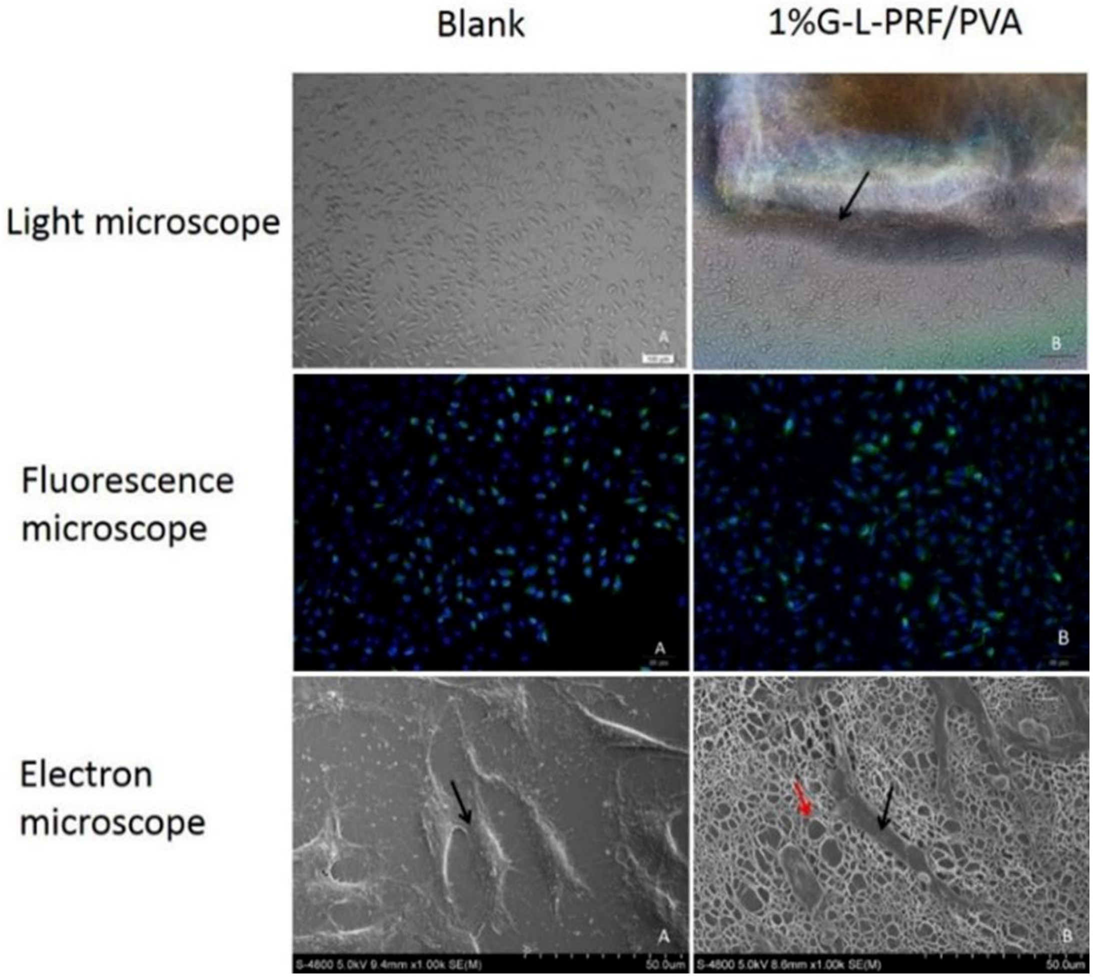

Figure S1. The L929 adhere to the dressing surface. Light microscope:(A) Blank control group, L929 were triangular and spindle shaped, (B)1%G-L-PRF/PVA group, the black arrows showed L929 at the junction. (bar = 100μm); Fluorescence microscope: (A) Blank control group, cell morphology is normal and clear back ground, (B)1%G-L-PRF/PVA group, cell spread out completely and the background blurred. (bar = 50μm); Electron microscope: (A) Blank control group, the cells were normal and fully spread, (B) 1%G-L-PRF/PVA group, morphologically normal cell adhesion with a porous dressing surface. (red arrow: dressing; black arrow: cell)

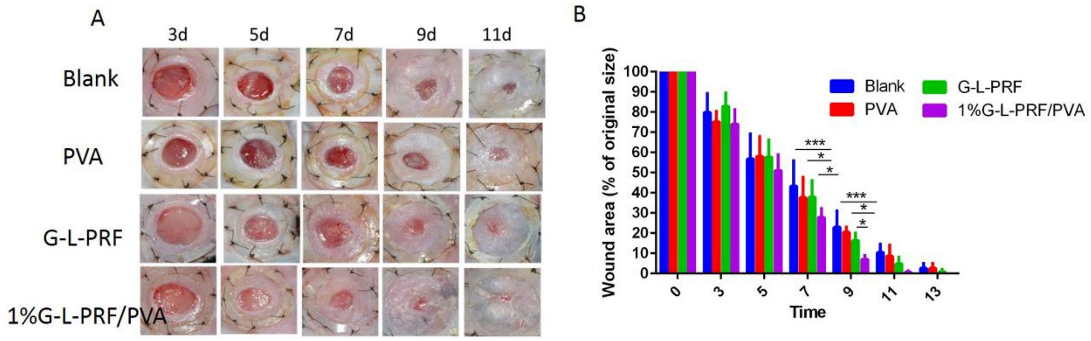

Figure S2. (A) Macroscopic observations of full-thickness acute wounds treated with PVA, LPRF, 1%G-L-PRF/PVA and Blank group for 3, 5, 7, 9 and 11days. (B) The size of the wound area of mice was measured by the Image-Pro Plus 6.0 software.

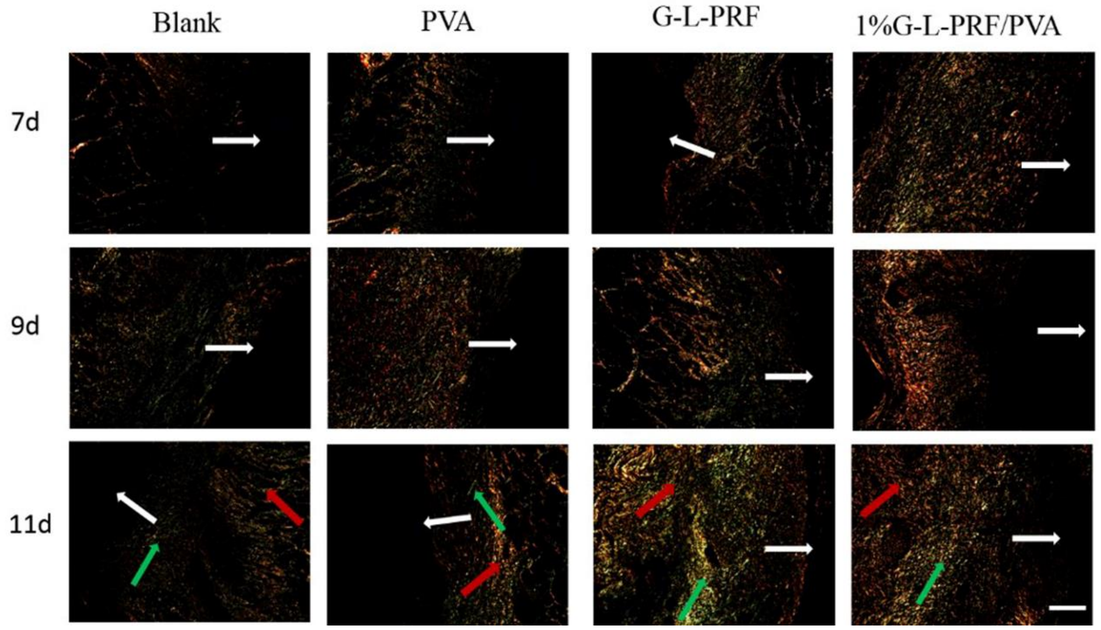

Figure S3. Picro-Sirius red stains images of sections after treated with four group under polarized light microscopy at different time points (bar = 100um) Red arrow, type I collagen, the brightly birefringent red and yellow, Green arrow, type III collagen, the weakly birefringent green fibers. White arrow, epithelial orientation.
